# Regulation of Microalgal Photosynthetic Electron Transfer

**DOI:** 10.3390/plants13152103

**Published:** 2024-07-29

**Authors:** Yuval Milrad, Laura Mosebach, Felix Buchert

**Affiliations:** Institute of Plant Biology and Biotechnology, University of Münster, Schlossplatz 8, 48143 Münster, Germany

**Keywords:** photosynthesis, microalgae, electron transfer regulation

## Abstract

The global ecosystem relies on the metabolism of photosynthetic organisms, featuring the ability to harness light as an energy source. The most successful type of photosynthesis utilizes a virtually inexhaustible electron pool from water, but the driver of this oxidation, sunlight, varies on time and intensity scales of several orders of magnitude. Such rapid and steep changes in energy availability are potentially devastating for biological systems. To enable a safe and efficient light-harnessing process, photosynthetic organisms tune their light capturing, the redox connections between core complexes and auxiliary electron mediators, ion passages across the membrane, and functional coupling of energy transducing organelles. Here, microalgal species are the most diverse group, featuring both unique environmental adjustment strategies and ubiquitous protective mechanisms. In this review, we explore a selection of regulatory processes of the microalgal photosynthetic apparatus supporting smooth electron flow in variable environments.

## 1. Introduction

### 1.1. The Diversity of Microalgal Oxygenic Photosynthesis

**Figure 1:**
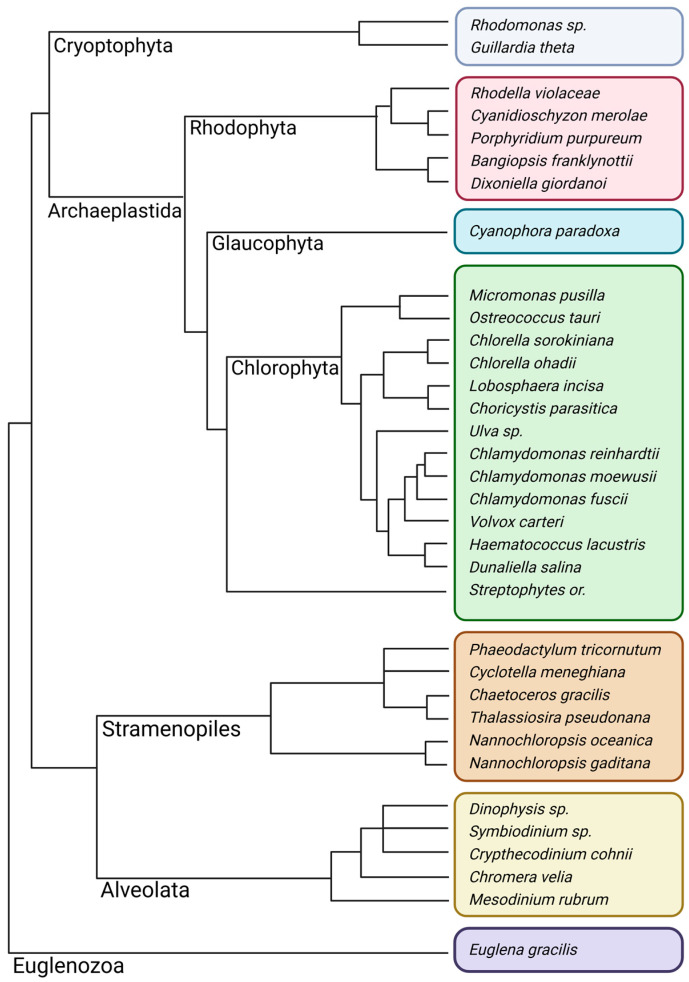
Simplified phylogenetic tree of microalgal groups. The latter are further subdivided in representative species that are partially covered in this review: Cryoptophyta (gray), Rhodophyta (red), Glaucophyta (cyan), Chlorophyta (with the addition of Streptophytes as reference group, green), Stramenopiles (orange), Alveolata (yellow), and Euglenozoa (purple). The illustration was created using Biorender.com.

Photosynthesis is a relatively ancient development of life on earth that uses light to capture CO_2_ via Ribulose-1,5-bisphosphate carboxylase/oxygenase (RuBisCO) activity. This review will focus on the most successful type of photosynthesis which is oxygenic and uses water as an electron donor for CO_2_ fixation in the Calvin Benson Bassham (CBB) cycle. It was initially developed in proto-cyanobacterial organisms which are dated as far as 2.3 Ga [1], with the earliest fossil findings pointing to 1.9 Ga [2]. As of now, there is an agreement that all oxygenic photosynthesizers originate from a single lineage of organisms, which possessed both type I and II photosynthetic reaction centers (aka PSI and PSII) [3,4,5]. These proto-cyanobacteria later evolved to the current day cyanobacteria to eventually engage in an endosymbiosis event (or events, see [6,7,8]), giving rise to the O_2_-producing plastids occurring in the lineage of Archaeplastida which includes green and red algae as well as Glaucophytes, but also in other domains such as Stramenopiles (e.g., diatoms) and Alveolata (e.g., dinoflagellates and Chromerida) [9]. As a very diverse group, microalgae can be found virtually everywhere, conquering both aquatic and terrestrial habitats including soil, aeroterrestrial and epiphytic habitats by developing unique adaptations [10]. The combination of a relatively short life cycle paired with a large eukaryotic genome, around 20–150 Mb (excluding exceptions [11]), might have helped microalgae to succeed in a competitive environment. Unsurprisingly, species of the same genus, such as *Chlorella*, were found in completely different environments—from Antarctic oceans [12,13] to Mediterranean deserts [14]—and in many cases feature distinct gene expression patterns in response to their habitat and the associated stress types [15], yet holding little genomic variation. On the other hand, the phenotypic expression of different algal lineages of similar habitats exhibits such converged traits that previous classification attempts led to a grand mix-up of genetic lineages [16]. In this review, we will shed light on the regulation of electron transfer processes that generate a transmembrane electrochemical proton gradient, also referred to as proton motive force (*pmf*). Our focus will be on how the *pmf* is fine-tuned for sustained photosynthetic productivity and how environmental adaptations altered these regulatory processes in different microalgae. However, functional microalgal photosynthesis research is entangled with research on other phototrophs since conserved fundamental processes are involved, such as energy stabilization upon water splitting in the oxygen-evolving complex (OEC). To fully cover how the *pmf* is regulated, this review will also lean on extrapolated knowledge derived from other photosynthetic domains. We will provide an overview of unique aspects of photosynthesis regulation in a selection of microalgal examples, acknowledging that covering the entirety of microalgal diversity will be beyond the scope of this review. To illustrate the heterogeneity of the term ‘microalgae’, we included a simplified phylogenetic tree (Figure 1, based on recent studies [9,17,18]), presenting the most relevant model organisms of eukaryotic microbial phototrophs.

### 1.2. The Oxygenic Photosynthetic Apparatus

**Figure 2:**
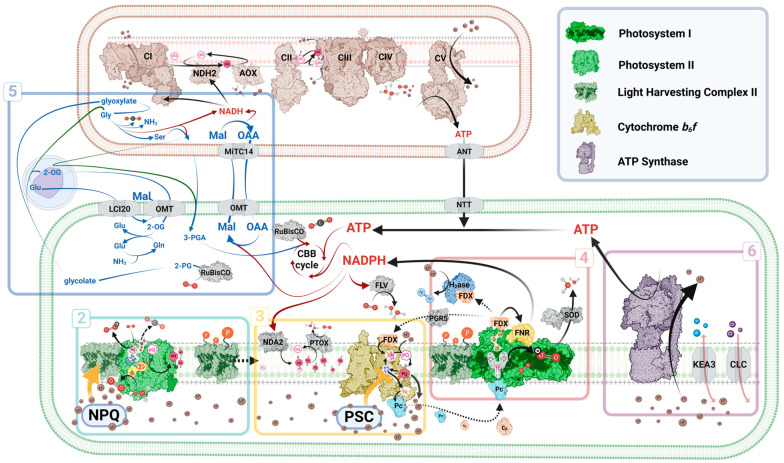
Schematic overview of photosynthetic electron transfer and regulatory processes. The numbered boxes refer to the sections of this review, mainly located within the chloroplast (green compartment; adhering to a green lineage blueprint), but also extending to the cytosol, peroxisomes (round object), and mitochondria (brown compartment). The green lines in box five represent vascular plant pathways. 2-OG: 2-oxoglutarate; 2-PG: 2-phosphoglycolate; 3-PGA: 3-phosphoglycerate; ATP: adenosine triphosphate; ANT: ATP and ADP translocases; AOX: alternative oxidase; b_6_f: cytochrome b_6_f complex; C: respiratory complex; c_6_: cytochrome c_6_; CBB: Calvin–Benson–Bassham; CEF: cyclic electron flow; CLC: Cl^-^ channel; FDX: ferredoxin; FNR: FDX:NADP^+^ oxidoreductase; FLV: flavodiiron protein; Gln: glutamine; Glu: glutamate; Gly: glycine; H_2_ase: hydrogenase; KEA3: K^+^ Exchange Antiporter 3; LCI20: low-carbon-inducible20; Mal: malate; NAD(P)H: reduced nicotinamide adenine dinucleotide (phosphate); MiTC14: mitochondrial substrate carrier protein 14; NDH: NAD(P)H dehydrogenase; NPQ: non-photochemical quenching; NTT: nucleoside triphosphate transporter; OAA: oxaloacetate; OMT: 2-OG/Mal translocator; Pc: plastocyanin; PGR5: proton gradient regulation 5 polypeptide; PSC: photosynthetic control; PSI/II: photosystem I/II; PQ: plastoquinone; PTOX: plastid terminal oxidase; RuBisCO: Ribulose-1,5-bisphosphate carboxylase/oxygenase; Ser: serine; SOD: Superoxide dismutase; UQ: ubiquinone. The illustration was created using Biorender.com.

In all oxygenic photosynthesizers, the *pmf* across the thylakoid membrane is constituted of two components: chemical (osmotic H^+^ gradient, ΔpH) and electric (membrane potential, ΔΨ). The electrons which are released during water oxidation (at the OEC of PSII) reduce a plastoquinone (PQ) molecule, situated in the acceptor side of PSII (Q_B_), converting it to plastoquinol (PQH_2_). As PQH_2_ diffuses within the membrane, it can reduce the cytochrome b_6_f complex (Cyt*b_6_f*) and by doing so, increase the capacity of *pmf* generation [19,20]. The electron transfer between Cyt*b_6_f* and PSI is then mediated by either plastocyanin (Pc) or cytochrome *c_6_* (Cyt*c_6_*). This variation originates from the altered metal cofactors and their environmental abundance, which in some cases determines the expression levels of Pc (containing a copper atom) and Cyt*c_6_* (containing an iron–heme cofactor) [21]. Some lineages, such as red algae, have lost the genes encoding Pc, while other lineages such as Charophytes and the derivative lineage of land plants almost exclusively rely on it. These lineages were thought to have completely lost the genes encoding Cytc_6_, although recent studies discovered Cytc_6_ orthologs that are still poorly characterized (e.g., Cytc_6A_ and Cytc_6B_) [22]. Adequately, these adaptations also triggered alterations of the interacting residues, situated on the PSAF loop of PSI, in both the green lineage during the transition to land [23] and across other photosynthetic lineages [24,25,26]. Following Pc/Cytc_6_ diffusion towards and reduction of photo-oxidized PSI, the energy stored within its excitation is channeled to the three [4Fe–4S] centers (F_X_, F_A_, F_B_). PSI then most prominently reduces ferredoxin (FDX), which is a small soluble electron carrier [27,28] that mediates a plethora of redox reactions, such as NADPH production via FDX:NADP^+^ oxidoreductase (FNR) [29,30]. The photo-reduced [2Fe-2S] cluster of FDX feeds into diverse redox carrier pools, such as thioredoxins and thioredoxin-like proteins [31]. Broadly, these processes are fine-tuned by an intricate regulatory network, aiming to maintain a proper *pmf* which allows bioenergetic membranes to engage in chemiosmosis via ATP synthase (F_O_F_1_) [32]. In Figure 2, we present a schematic illustration of the photosynthetic apparatus, based on green microalgal physiology. The boxes highlight the sections covered in this review, comprising a selection of the latest findings in the field.

## 2. Maintaining Proper Water Splitting

### 2.1. Spatial Separation of the Thylakoid Membrane

To date, most of our knowledge on the regulatory aspects of membranal organization is based on land plant thylakoids, but microalgal studies are on the rise owing to recent advances in cryo-focused ion beam milling and cryo-electron tomography [33,34,35]. Accordingly, structural properties shared between plant and green microalgal membranes are the division into appressed (aka grana) and non-appressed (aka stromal lamellae) domains [36]. However, unlike the 4–20 layered cylinders that form vascular plant grana stacks [37], thylakoid stacking is less pronounced in nonvascular plants [38] and even further reduced in green microalgae such as *Chlamydomonas reinhardtii* [33,34,39]. While red algae and Glaucophytes have unstacked thylakoids likely due to their phycobilisomes, appressed membrane bands are usually found in secondary and tertiary plastids of Stramenopiles, Haptophytes, dinoflagellates, and Cryptophytes. The distinction between appressed and non-appressed membranes results in a lateral heterogeneity among the distribution of photosynthetic membrane protein complexes: PSII tends to be localized in membrane stacks, while PSI and F_O_F_1_ reside in unstacked membranes [40]. The Cyt*b_6_f* is present in both domains and laterally mobile [41,42]. Lateral heterogeneity might help to separate PSI from PSII to prevent energy spillover [43]. The latter may be differently controlled within red algal membranes, being predominantly non-appressed with homogeneously distributed complexes [39]. Here, a row-like organization of phycobilisome-covered PSII might control spillover efficiencies [44]. Furthermore, a feedback mechanism was recently postulated that connects grana stacking with the *pmf* in the form of light-dependent luminal cation concentration [45]. Indeed, it was shown that in the absence of adequate grana stacking, the stress adaptability of vascular plants was diminished [46]. Accordingly, such de-stacking might deregulate the spatial separation of the two photosystems and/or diffusion of the electron carriers. Data from vascular plants suggest that the spatial separation of the two photosystems limits electron flow to some extent due to the diffusion of PQH_2_ and Pc/Cyt*c_6_*, and at least the luminal diffusion was reported to be dependent on the intermembrane space [47,48]. In land plants, the diffusion of PQ/PQH_2_ within the membrane was shown to be regulated by the formation of super-complexes and the viscosity of the membrane [49]. Microalgal studies in this context are scarce, but a functional link of PQ/PQH_2_ diffusion and/or membrane viscosity to PSII repair was recently proposed [50]. Interestingly, one converged adaptation to cold environments, such as in Antarctica, is the desaturation of fatty acids. This was demonstrated to increase the fluidity of the thylakoid membrane [51]. Some green algal species, such as the Antarctic *Chlamydomonas raudensi* [52] but also the temperate *Lobosphaera incisa* [53], contain polyunsaturated fatty acids which greatly increase lipid fluidity. Accordingly, these adaptations were shown to increase the mobility of PQ/PQH_2_ molecules in the membrane, and were postulated to play an essential role in enabling adequate gas exchange [54]. By coping with such restraints, these branches of the photosynthetic lineage conquered some of the harshest environments, which seem to be inhabitable to most other oxygenic phototrophs. Similar adaptations were reported to play a key role in regulating electron flux rates in Stramenopiles, such as *Phaeodactylum tricornutum*, where the saturation state of thylakoid fatty acids increased in correlation with PQ pool oxidation, promoting PQ/PQH_2_ diffusion [55]. Taken together, microalgae may provide versatile solutions to establish photosynthetic resilience, and combined efforts of ultrastructural and functional studies will be required to elucidate the diversity of microalgal adaptations.

### 2.2. Intrinsic PSII Regulation

Oxygenic photosynthesis depends on the common reaction of water oxidation by the OEC, situated in PSII (for a full review on the history of PSII discoveries, see [56]; for a detailed review covering PSII function, see [57]). Briefly, excited electrons are channeled to a semi-quinone, located in the permanent Q_A_ site of PSII, from which they are transferred to a PQ molecule, situated in the Q_B_ site (aka PSII acceptor side [58]). One of the main hazards related to PSII activity is the formation of singlet oxygen at the P680 reaction center, being generally very destructive for living organisms [59,60]. Therefore, PSII is one of the most regulated complexes in the chloroplast. Damaged reaction centers are routinely degraded, while the chassis of the peripheral subunits stays untouched during swift reassembly [61]. The rate at which this chain operates depends on many factors. The antenna size and configuration determine the amount of energy that enters the system (see Section 2.3). In addition, it was shown that low HCO_3_^−^ availability in the lumen can inhibit the activity of the OEC [62], as can increasing concentrations of ascorbate [63,64]. One way PSII senses downstream bottlenecks is by the availability of PQ, which reflects changes in Cyt*b_6_f* activity (see Section 3.1). In the absence of available PQ, electrons fail to exit the Q_A_ site and therefore perform a back reaction potentially ending up in singlet oxygen formation [65]. It was postulated that at this point, Q_A_ can reduce O_2_ to relieve the redox pressure on the center. However, when O_2_ is not available due to increased mitochondrial activity or external hypoxia, the mode of operation alters [66]. In mature PSII complexes, the non-heme iron, situated between the Q_A_ and Q_B_ sites, is in complex with an HCO_3_^−^ ion [45,67,68,69]. HCO_3_^−^ is incorporated during PSII maturation replacing a Glu sidechain complexed with non-heme iron [68]. Recent observations showed that a similar, yet analogous maturation process precedes the incorporation of the OEC in cyanobacterial systems [70]. This could represent a universal photoprotective strategy during PSII maturation to limit singlet oxygen formation. Indeed, it was reported that replacing HCO_3_^−^ with glycolate decreased PQ reduction and boosted O_2_ reduction at the Q_A_ site [71]. Moreover, the output of electrons into the PQ pool was observed to be decreased in such conditions [62,72,73], leading some authors to postulate that the redox change alters the electron flow pathway to a PSII-cyclic mode [74,75,76,77]. This mechanism might involve a yet to be characterized Q_C_ site [78,79] and/or an intrinsic route involving cytochrome *b*_559_ [80,81,82]. In any case, these redox changes within PSII were associated with an increased or highly variable *pmf* formed across the thylakoid membrane.

### 2.3. Rapid Adjustments of Light-Harvesting Capacity

Light-harvesting determines the energy input into the photosynthetic electron transport chain, so that fine-tuning of light-harvesting is vital to balance energy supply with metabolic demands and to diminish the production of harmful reactive oxygen species (ROS), such as the aforementioned singlet oxygen. Energy-dependent (qE) non-photochemical quenching (NPQ) mediates the thermal dissipation of excess excitation energy. Thereby, the effective photosynthetic contribution of PSII can be fine-tuned on a short time scale. In the green algal model species *Chlamydomonas reinhardtii*, qE depends on light harvesting complex stress-related 3 (LHCSR3) [83] and to a minor extent on LHCSR1 [84]. PSBS, the main qE catalyst in vascular plants [85], likely contributes to the structural reorganization occurring during qE induction as well as a minor LHCSR-independent qE component [86,87,88,89]. Likewise, LHCSR3 and PSBS facilitate qE during the first phase of the photoprotective response in the green microalga *Haematococcus lacustris*, whereas during the second phase, optical shielding by astaxanthin accumulated in the mature hematocysts predominates [90]. The molecular docking site of LHCSR at PSII-LHCII is elusive. In *Chlamydomonas reinhardtii*, PSBR is required for efficient LHCSR3 binding to PSII-LHCII [91,92], and LHCB5 [93,94] as well as LHCBM1 [95] have been reported to be essential for qE. LHSCR proteins bind pigments and sense the lumen pH via protonatable residues [96,97,98]. This creates a regulatory feedback loop between electron transfer and light-harvesting, since the lumen pH reflects the redox state of the electron transport chain: Lumen acidification triggers protonation-induced conformational changes of LHCSR [99], leading to a functional switch of the LHCII antennae system from a light-harvesting to an energy-dissipating state [100].

Among other factors, LHCSR and PSBS accumulation in *Chlamydomonas reinhardtii* depends on light and intracellular CO_2_. Thus, LHCSR and PSBS levels are a function of excitation energy availability and metabolic sink capacity: Expression of both *LHCSR3* [83,97,98,101] and to a lesser extent *LHCSR1* [84,102] is induced in response to high light, while *PSBS* expression occurs transiently following the onset of high light [87,88,89]. *LHCSR1* and *PSBS* expression is primarily promoted in response to UV light, a condition in which LHCSR3 accumulates to a lesser extent [86,103,104]. Interestingly, expression of *LHCSR* and *PSBS* in response to light is differentially regulated in the green alga *Haematococcus lacustris*, exemplifying the diversity of qE regulation even within the group of green microalgae [105]. Furthermore, *LHCSR3* and *PSBS* expression in *Chlamydomonas reinhardtii* is induced in response to low CO_2_ levels via a shared EEC enhancer sequence motif [87,106,107]. Notably, the carbon-concentrating mechanism (CCM) master regulator CIA5/CCM1 [108,109] promotes *LHCSR3* expression and slightly induces *PSBS* expression, even in the absence of light, whereas it inhibits LHCSR1 accumulation [104,110]. Light- and CO_2_-dependent signaling partially intertwines [111], resulting in a coregulation of photoprotection- and CCM-related genes [104,110,112]. The differential expression patterns of *LHCSR3* and *LHCSR1* in response to light and CO_2_ signals suggest these proteins may play complementary roles in balancing photoprotection with light-harvesting efficiency [113].

Moreover, lumen acidification induces the two-step enzymatic de-epoxidation of violaxanthin to antheraxanthin and zeaxanthin reversibly associated with LHCII [114]. Although in *Chlamydomonas reinhardtii* a contribution of zeaxanthin and/or LHCII aggregation to qE has been previously discarded [115], other recent studies report the existence of a zeaxanthin-dependent qE component [98] as well as the capability of aggregated LHCII trimers to mediate qE via LHCBM1 and LHCBM5 [116,117]. Intriguingly, *Chlamydomonas reinhardtii* features an atypical violaxanthin de-epoxidase, being located to the stromal face of the thylakoid membrane [118] and not relying on ascorbate as a reductant [119]. In contrast, in the green alga *Chlorella vulgaris,* qE clearly depends on zeaxanthin accumulation mediated by a plant-like violaxanthin-de-epoxidase [120]. In *Chlamydomonas reinhardtii* and other green algae as well as algae containing secondary green plastids, an additional xantophyll cycle involving lutein and loroxanthin operates on longer time scales, similar to the lutein–epoxide/lutein cycle in plants [121].

In microalgal species containing secondary red plastids, qE unambiguously relies on the xanthophyll cycle. In *Chromera velia* (Alveolata), qE is induced by a fast de-epoxidation of violaxanthin to zeaxanthin in response to lumen acidification [122]. In Stramenopiles such as *Nannochloropsis gaditana* [123] and *Nannochloropsis oceanica* [124,125], qE involves LHCX proteins quenching LHCs at both photosystems as well as zeaxanthin-dependent quenching of LHCII. Likewise, in other Stramenopile model species such as *Phaeodactylum tricornutum,* LHCX proteins play a major role in qE [126], with different isoforms being expressed in response to a multitude of abiotic factors and mediating distinct quenching mechanisms [127,128,129,130]. Notably, unlike LHCSR proteins, LHCX proteins are not involved in sensing the lumen pH, while diadinoxanthin/diatoxanthin binding is essential for qE induction [131,132]. The modulation of qE in diatoms occurs via activity regulation of both xanthophyll cycle enzymes, diadinoxanthin de-epoxidase and diatoxanthin-epoxidase, mediating the single-step conversion between diadinoxanthin and diatoxanthin [133,134].

Evolutionarily earlier branching microalgae sustained both phycobilisomes (for recent reviews see [135,136,137]) and light-harvesting antenna proteins, concomitant with diverse photoprotective mechanisms. In the Cryptophyte *Rhodomonas salina*, qE is independent of a xanthophyll cycle, but involves the protonation of light-harvesting antenna proteins [138]. Being independent of both a xantophyll cycle and a ΔpH, qE in the Rhodophyte *Dixoniella giordanoi* has been attributed to a functional disconnection of phycobilisomes from PSII [139]. In contrast, pH-induced qE in other Rhodophytes such as *Porphyridium purpureum* occurs at the PSII core antenna and likely involves a yet to be identified qE effector protein [140].

qE genetic regulation in response to light intensity and quality proceeds via photoreceptor-mediated anterograde signaling. In *Chlamydomonas reinhardtii*, the blue-light photoreceptor phototropin (PHOT) controls *LHCSR3* induction [141,142]: Upon blue-light sensing by the PHOT-LOV domains, signal transduction is initiated via the PHOT-kinase domain and results in a derepression of *LHCSR3* transcription via inhibition of the involved ubiquitin ligase complex [143,144,145]. Intriguingly, blue-light sensing in the Stramenopile *Phaeodactylum tricornutum* proceeds similarly via the LOV domain of AUREO1c. However, AUREO1c directly activates *LHCX* transcription via a bZIP domain, enabling a more rapid induction of gene expression. These findings illustrate a case of convergent evolution between green algae and diatoms in terms of signal perception, with diverging downstream gene regulatory processes [146].

### 2.4. State Transitions Redistribute Energy Conversion Efficiencies

State transition-dependent NPQ (qT) is realized within minutes based on a redistribution of excitation energy between the two photosystems in response to the redox state of the PQ/PQH_2_ pool. If PQ reduction prevails over PQH_2_ oxidation, a transition from state I to II is induced: A mobile fraction of LHCII is phosphorylated and dissociates from PSII to reversibly associate with PSI, thereby readjusting the relative absorption cross-section and re-establishing the redox poise of the photosynthetic electron transport chain [147,148]. As already reported by early studies, the relative absorption cross-section in the green algal model species *Chlamydomonas reinhardtii* is modulated by 50–80% [149,150], promoting both photosynthetic efficiency in low light and photoprotection in high light [101,151,152]. In *Chlamydomonas reinhardtii*, redox-induced phosphorylation of LHCII is mediated by the membrane-associated Ser-Thr kinase STT7 [153], while dephosphorylation of LHCII occurs constantly via the PP2C-type phosphatases PPH1 and PBCP [154]. So far, high-resolution structures of algal state transition complexes have been obtained from *Chlamydomonas reinhardtii* [155,156] and the primordial green alga *Ostreococcus tauri* [157]. In both PSI-LHCI-LHCII/LHCP structures, association of one LCHII/LHCP trimer involves an N-terminal phosphorylated Thr residue of LHCII/LHCP and PSAH/PSAL/PSAO. The overall number and positioning of LHCII/LHCP trimers however differs between the two species: In *Chlamydomonas reinhardtii*, binding of a first LHCII trimer is facilitated by LHCBM1 phosphorylated at Thr27, whereas association of a second LHCII trimer relies on interactions of LHCBM5 phosphorylated at Thr33 with PSAH/LHCA2 [156]. In *Ostreococcus tauri*, three LHCP trimers associate with PSI-LHCI between LHCA6 and PSAK [157].

## 3. Regulations Revolving around Cytochrome *b_6_f*

### 3.1. Photosynthetic Control Diminishes Cytochrome b_6_f Activity to Protect PSI

PQH_2_ oxidation at the luminal Q_o_ site of Cyt*b_6_f* is pH-dependent and limits the rate of photosynthetic electron transfer [158,159,160,161]. Thus, in addition to light-harvesting, lumen acidification modulates electron flow, a mechanism termed photosynthetic control [162,163]. On a molecular level, it has been proposed that low lumen pH results in the protonation of a Rieske ISP His residue ligating the [2Fe-2S] cluster [164], impacting the switching rate of Rieske ISP between the distal and the proximal position and thereby decelerating PQH_2_ oxidation. An alternative mechanistic model was postulated [165], supported by functional cytochrome *bc*_1_ complex studies from respiratory membranes [166,167]: A disulfide of unknown function, adjacent to the Rieske [2Fe-2S] cluster, influences the redox midpoint potential upon enzymatic formation of the luminal disulfide [168] and dithiol [169], respectively. Photosynthetic control mutants are available with alterations in the proximity of the [2Fe-2S] cluster and the disulfide. The substitution of a conserved Rieske ISP Pro with Leu hypersensitizes Cyt*b_6_f* for ΔpH in *Arabidopsis thaliana* [170,171] and *Chlamydomonas reinhardtii* [172]. This amplification of photosynthetic control presumably stems from a shift of the pK_a_ and/or the redox potential of Rieske ISP. However, on first approximation, the Rieske ISP point mutation was less efficient in slowing down the electron transfer chain in *Chlamydomonas reinhardtii*. One possible explanation could be a unique algal lumen pH during the induction of photosynthesis, hardly reaching a critical acidification at which the Rieske ISP point mutation excessively limits photosynthesis in *Arabidopsis thaliana*. There is further evidence, partially derived from photosynthetic control experiments, that microalgal photosynthesis operates at different lumen acidification levels. When compared with green algae, which show half-maximal inhibition at pH 6.3 [173], it appears that photosynthetic control of Cyt*b_6_f* in diatoms is shifted towards lower pH values of around 4.7 [174]. This might as well coincide with a similar lumen pH shift to induce NPQ in diatoms [174] and could point to variances in the H^+^/ATP ratio imposed by the F_O_F_1_-ATP synthase (see Section 6.1).

### 3.2. Cyclic Electron Flow Maintains ATP Levels in Relation to NADPH Production

Cyclic electron flow (CEF) recycles electrons from the PSI acceptor side to upstream components of the electron transport chain [175]. In this way, CEF impacts the *pmf* and establishes a key regulatory feedback loop. CEF provides additional ATP that is independent from NADPH production and can be used for CO_2_ fixation, which was shown in green algae to require an NADPH/ATP ratio of 2:3 [176]. Moreover, CEF sustains CCM, photorespiration and other metabolic processes [177,178]. It also induces ΔpH-dependent photoprotective mechanisms such as qE and photosynthetic control [179]. Early inhibitor studies with *Chlamydomonas reinhardtii* [180] and isolated *Pisum sativum* chloroplasts [181] distinguished two distinct CEF pathways: antimycin A-insensitive CEF involving NAD(P)H dehydrogenase complexes (NDH-dependent CEF) and antimycin A-sensitive CEF relying on an FDX-PQ reductase activity (FQR-dependent CEF). In *Chlamydomonas reinhardtii* and most other green algae, NDH-dependent CEF is facilitated by a monomeric type II NDH complex (NDA2) [182,183]. NDA2 exhibits two Rossmann-fold domains mediating FMN and NAD(P)H binding, and the enzyme is located at the stromal side of the thylakoid membrane [184]. Recombinantly overexpressed NDA2 preferentially oxidizes NADH [185], implying that NDA2 might rely on a transhydrogenase for substrate supply in vivo [186]. NDA2 exhibits two EF hands hinting at a potential Ca^2+^-dependent regulation [187]. Furthermore, NDA2 has been detected phosphorylated in reducing conditions [188]. In *Chlamydomonas reinhardtii*, the proportion of light-dependent PQ reduction derived from NDH-dependent CEF is negligible [189]. However, NDA2 accesses NAD(P)H from endogenous carbon sources for PSII-independent H_2_ production [190,191,192]. Moreover, NDA2 mediates light-independent PQ reduction as the first step of chlororespiration [193], being completed by O_2_ reduction via PQH_2_-terminal-oxidase (PTOX) as a second step [194,195,196,197]. Chlororespiration is a part of cellular dark metabolism and has been suggested to poise the PQ/PQH_2_ pool for the onset of illumination [193,198]. In species that do not rely on monomeric type II NDH complexes, the NDH-dependent CEF pathway is electrogenic. Therefore, chlororespiration could also sustain membrane polarization in the dark. Furthermore, it was shown to be an important valve under restricting conditions, as demonstrated in starch deficient mutants of *Chlamydomonas reinhardtii* [199] or in nutrient-deprived *Ostreococcus* species [200]. In *Chlorella ohadii,* which was reported to be exceptionally resilient to high light exposure [201,202], chlororespiration was shown to play an important role in fast adaptations to high irradiance [203].

### 3.3. Ferredoxin-Plastoquinone-Reductase-Dependent Cyclic Electron Flow

FQR-dependent CEF is the predominant CEF pathway in the green algal model species *Chlamydomonas reinhardtii* [204,205]. Dating back to first experiments with isolated *Spinacia oleracea* chloroplasts [206,207], the molecular mechanism of antimycin A-sensitive CEF has not been elucidated yet. Interestingly, early inhibitor studies of FQR-dependent CEF in *Spinacia oleracea* and *Pisum sativum* hint at an involvement of Cyt*b_6_f* [208] and FNR [209,210,211]. Furthermore, studies in *Arabidopsis thaliana* [212,213] and *Chlamydomonas reinhardtii* [214,215,216,217,218] identified proton gradient regulation 5 (PGR5) and its interaction partner PGR5-like 1 (PGRL1) as factors implicated in antimycin A-sensitive CEF. FQR-dependent CEF appears to be functional in the absence of PGRL1 [189,219] and recent studies in *Chlamydomonas reinhardtii* imply that Cyt*b_6_f* may in fact represent the elusive FQR, with PGR5 being required for sustained stromal electron input [220,221], presumably via supporting the association of FNR with the thylakoid membrane [222]. Surprisingly, these CEF pathways seem to be missing under permissive conditions in *Euglena gracilis*, an organism containing secondary green plastids [223]. However, this photosynthetic alga is known to display a robust metabolism which might compensate the absence of CEF, possibly by having acquired genes from a multitude of photosynthetic organisms [224]. Interestingly, two of the Cyt*b_6_f* subunits usually encoded in the chloroplast genome of photosynthetic eukaryotes that display CEF, Cytochrome-*f* (PetA), and subunit-IV (PetD) are exported to the nuclear genome of *Euglena gracilis* [225]. This raises the question to which extent certain euglenoid Cyt*b_6_f* functions have been sacrificed during this peculiar evolutionary history. Notably, the secondary red plastids of diatoms such as *Phaeodactylum tricornutum* and *Thalassiosira pseudonana* feature PGR5/PGRL1 homologues [226,227] potentially implicated in CEF [228]. Despite low constitutive CEF rates reported in most diatoms [229], CEF may play an essential role in response to stress conditions [230].

In the green algal model species *Chlamydomonas reinhardtii*, a PSI–Cyt*b_6_f* supercomplex potentially mediating CEF has been isolated from conditions where it is required to alleviate stromal reducing pressure [188,231,232,233,234]. Besides PGRL1 and FNR, PSI–Cyt*b_6_f* included CAS, ANR1, and PETO as potential further actors of FQR-dependent CEF: Being a Ca^2+^-sensing protein involved in the regulation of photoprotection- and CCM-related gene expression [107,214,235], CAS has been suggested to facilitate Ca^2+^-dependent activity regulation of FQR-dependent CEF [232,236]. ANR1 has been proposed to sense the PQ/PQH_2_ redox state or the *pmf* and the algal Cyt*b_6_f* subunit PETO has been hypothesized to sense the stromal redox state [237]. Alternatively, ANR1 and PETO have been postulated to mediate FDX binding to Cyt*b_6_f* [238]. In addition, the interaction interface of PETO with the Cyt*b_6_f* encompasses several STT7-dependent phosphorylation sites and both ANR1 and PGRL1 have been observed phosphorylated as well [188,221,238,239], implying a phosphorylation-dependent regulation of FQR-dependent CEF. Although in the green algal model the molecular mechanisms are still elusive, even less details are available in other microalgal groups, and it remains to be seen if these organisms also engage specific auxiliary CEF proteins or show phosphorylation-dependent fine-tuning of the involved players.

## 4. PSI Acceptor Side Downstream Processes

### 4.1. Algal Response to Excess Light Bursts

Microalgae face an ever-changing environment as many of them live in murky ponds or oceans. As mentioned above, they experience and safely deal with abrupt light fluctuations in their habitats. Microalgae not only rely on light supply to fix carbon and store metabolites, they also regulate their life cycles in a light-dependent manner [240]. Yet, many of their stress responses and regulatory processes revolve around the photosystems. In contrast to PSII, the PSI core is very inert and cannot go through an efficient repair process [241,242,243]. Therefore, once the P700 reaction center is excited, it has to be relieved or else the entire complex will degrade while reducing O_2_ to form potent radical species [59]. Acceptor side limiting conditions can generate a severe bottleneck for electron transport out of the PSI reaction center, so that photosynthetic organisms have developed several protective valves to minimize the lifetimes of excited states within the complex. These strategies sustain the electron transfer activity and the associated *pmf* generation for ATP synthesis. The most imminent sink that PSI has is situated within the complex itself: O_2_ molecules can be reduced by the phylloquinones of PSI in a process termed ‘Mehler reaction’, yielding superoxide anion radicals [244]. This can take place concomitant with NADPH formation [245] and could be regarded as the default release valve, since owing to the conserved structure of PSI, Mehler reactions occur throughout all photosynthetic organisms [246]. For instance, Mehler reactions have been reported to play a central role in photoprotection of coral-symbiont species (*Symbiodinium* sp., dinoflagellates) [247]. Importantly, the extent of superoxide anion radical formation due to Mehler reactions is far greater than the amount of ROS formed elsewhere in the photosynthetic apparatus under various conditions [248]. Yet, unlike other O_2_ scavenging processes, the Mehler reaction involves only a single electron transfer step and no intermediate complex is formed, i.e., superoxide anion radicals are released directly to the stroma. However, this type of ROS is considered to be less damaging [249], since superoxide anion radicals are rapidly converted into hydrogen peroxide by superoxide dismutase [250]. The formed hydrogen peroxide can either induce gene expression [251,252,253] or is further detoxified to water by catalase, completing the ‘Water–water cycle’ [254].

### 4.2. Oxygen Coupled Scavengers Avert Excessive Reduction and Serve as Electron Sink

Upon the reduction of FDX, the oxidized PSI acceptor side [4Fe–4S] clusters are prepared for the next photoreduction. It is for that reason that FDX plays a crucial role in maintaining the functionality of the electron transport chain. However, when the light energy input surpasses the capacity of downstream production (and consumption) of metabolites with limited pool sizes, such as NADPH, the cells will direct excess energy towards other pathways. Evidently, many of these pathways are O_2_ scavengers, which reduce O_2_ to hydrogen peroxide. These processes may be mediated by FDX itself [255], being referred to as pseudo-Mehler reactions. However, other O_2_-reducing pathways consume NADPH and thus result in a dual benefit: First, they provide the oxidized substrate for FNR, and second, they help to adjust the NADPH/ATP ratio for the CBB cycle (in addition to FQR-dependent CEF mentioned in Section 3.3). One example is the activation of flavodiiron proteins (FLVs), being crucial for the stress response in microalgae [256,257,258] as well as photosynthetic organisms from other branches, excluding angiosperms [259]. Interestingly, many organisms hold at least two variants of these proteins, which are expressed differently under constitutive versus stress conditions. Some isoforms were found to be highly expressed in response to high light, in which the NADPH/ATP ratio is very high [259]. Moreover, increased NADPH levels would result in an additional reduction of the PQ pool by NDH-dependent CEF, which would in turn generate additional limitations at the PSII acceptor side and induce state transitions (see Section 2.4). Increased FLV expression was also observed under carbon limitations [259], in which high levels of NAPDH may promote the oxygenation reaction catalyzed by RuBisCO (see Section 5.1). The consumption of both NADPH and O_2_ in a single process seems to be the simplest logical path, as has been shown to be the case in a cyanobacterial system [260]. Other strategies include extended pyruvate or acetyl-CoA fermentation, which results in increased energy channeling to other organelles (see Section 5.2). Spread across oxygenic photosynthesizers and predating endosymbiosis, these pathways include pyruvate:NADP+ oxidoreductase (PNO) or pyruvate:ferredoxin oxidoreductase (PFO) as well as pyruvate formate–lyase (PFL) and aldehyde/alcohol dehydrogenase (ADHE) [261,262,263]. However, when microalgae experience anaerobiosis, due to excessive respiration or environmental conditions, the lack of O_2_ hinders PSII activation [66,72,264]. This poses a potential threat to the system, where the rapid onset of primary photochemistry would occur in the absence of immediate electron acceptors (PQ, FDX). As a response, algal gene expression alters and promotes a ‘brace for impact’ state. Notably, hydrogenase (H_2_ase), which acts as ‘rapid response valve’, is present in all unicellular photosynthetic branches, ranging from sulfur bacteria to algae [265,266,267]. Their assimilation in the eukaryotic lineages can be traced to different origins and was postulated to be the outcome of independent endosymbiosis events [268], and H_2_ase activity was identified in many Archaeplastidae species (excluding Mamiellophyceae and Streptophytes, which have lost the encoding genes), as well as Stramenopiles [263]. When activated, H_2_ase reduces two protons to molecular H_2_ in a reversible manner [269,270,271]. These enzymes are very sensitive to O_2_ and are only highly expressed under dark anaerobiosis [272]. When exposed to light bursts, algae evolve H_2_ at high rates which decrease once the system adjusts to shift into CO_2_ fixation mode [273]. A similar gene expression pattern is observed for the FQR-dependent CEF auxiliary protein anaerobic response 1 (ANR1), demonstrating that the purposeful competition between sustained H_2_ase activity [186,274,275,276] and NADPH production for CO_2_ fixation facilitates a smooth transition to O_2_ production in the light.

## 5. Inter-Organellar Interaction

### 5.1. Photorespiration and Dealing with a Nondiscriminatory RuBisCO

As mentioned above, oxygenic photosynthesis relies on a calculated lack of energy stored based on linear electron flow alone, being exemplified by the mismatched energy carrier ratio of ATP to NADPH required for CO_2_ fixation. Therefore, photosynthesis cannot be an isolated process to ensure cell survival but is interconnected with several metabolic pathways across organelles in eukaryotic species. Importantly, the modules plugged into the photosynthetic membranes evolved in an environment of high CO_2_ and low O_2_ concentrations—much different from today’s atmospheric levels. Accordingly, phototrophs are constantly facing consequences of nondiscriminatory O_2_-fixing RuBisCO reactions. Besides CO_2_ fixation, RuBisCO promiscuously reacts with O_2_ which produces the toxic intermediate 2-phosphoglycolate (2-PG) that inhibits several CBB enzymes [277]. To prevent the accumulation of this dead-end intermediate, 2-PG undergoes a series of enzymatic reactions to be recycled back into the CBB cycle intermediate 3-phosphoglycerate in a process called ‘photorespiration’ [278]. This recycling pathway uses ancient metabolic modules and requires about ten core enzymes which, in land plants, are located in chloroplasts, peroxisomes, and mitochondria. Photorespiration accounts for a loss of CO_2_, NH_3,_ as well as energy in the forms of ATP and NADPH. However, this process is of tremendous importance, especially in terrestrial photosynthesis, as there is a 25% chance of the oxygenation reaction catalyzed by RuBisCO in a C_3_ leaf [279]. Overall, photorespiration fuels mitorespiration by forming NADH upon the CO_2_-releasing conversion of two Gly to Ser, but the pathway is less well-studied in marine phototrophs such as diatoms. This diverse group of microalgae possesses very efficient CCMs resulting in low photorespiration rates which limits further insights on metabolic shortcomings in the absence of 2-PG recycling [280,281]. This is better understood in green algae such as *Chlamydomonas reinhardtii*, in which photorespiration proceeds differently compared to land plants. Indeed, the alga seems to bypass the peroxisome to some extent. This might be linked to the fact that the number of peroxisomes is strongly dependent on the availability of reduced carbon in the growth medium of *Chlamydomonas reinhardtii* [282]. Moreover, the organelles are much more primitive and, like in many microalgae [283,284], catalase is not the typical peroxisomal marker known from vascular plants [285]. Accordingly, a photorespiratory bypass of the organelle might allow for a more reliable flux management. For instance [286], glyoxylate formation from glycolate occurs in the mitochondria (rather than the peroxisomes) through glycolate dehydrogenase (rather than glycolate oxidase). An unusual localization also applies to the penultimate photorespiratory step, where Ser stemming from the mitochondria is converted on the level of hydroxypyruvate reductases (HPR), of which the alga possesses an array of extra-peroxisomal isoforms [287,288]. Accordingly, HPR1 from *Chlamydomonas reinhardtii* is located in the mitochondria and its deletion has severe growth defects [288], whereas the isoform from *Arabidopsis thaliana* is located in the peroxisome and mutant plants display no noticeable phenotype [289,290]. This indicates that, despite the microalgal CCM to counter O_2_ fixation via RuBisCO, photorespiration is a vital process for unicellular phototrophs. Future studies in freshwater and marine models are required to clarify if there is a general dependency of photorespiration redundancy on CCM efficiency, i.e., if photorespiratory activities are recruited under specific conditions in microalgae.

### 5.2. Malate Shuttle Dissipates Plastidial Redox Pressure and Is Auxiliary to Photorespiration

The malate (Mal) shuttle, also referred to as Mal valve [291], can be regarded as another inter-organellar safety mechanism to lower the plastidial redox pressure in the stroma, thereby establishing metabolic connectivity with other cellular compartments. Unlike NAD(P)H, Mal is efficiently trafficking across organellar membrane barriers thanks to various Mal translocator and interconversion systems (reviewed in [292]). Best understood in land plants, the Mal shuttle comes in different flavors: An important player involves Mal dehydrogenases (MDH) which interconvert Mal with oxaloacetate (OAA) by coupling its reversible activity to the NAD(P)^+^/NAD(P)H pools [293]. Chloroplast, cytosolic, peroxisomal, and mitochondrial MDH are important contributors to the cellular redox landscape. MDHs provide substrates for the Mal/OAA translocators that can be found at least in chloroplast and mitochondrial membranes. The concerted action of MDH and Mal/OAA translocators connect ATP production via photophosphorylation and oxidative phosphorylation. By consuming NADPH in the chloroplast, Mal formation sustains electron transfer coupled to light-driven ATP synthesis, whereas mitochondrial OAA formation yields NADH to fuel mitochondrial electron transfer for oxidative phosphorylation. Mitochondrial ATP could be imported into the plastid via nucleoside triphosphate transporters and other pathways reviewed in [294]. While plant homologs are better understood (reviewed in [292]), only putative candidates are available to catalyze Mal/OAA exchange in the green algal model organism *Chlamydomonas reinhardtii*, i.e., the plastidial 2-oxoglutarate (2-OG)/Mal translocator (OMT1/2) and the mitochondrial substrate carrier protein 14 (MiTC14) [295]. The very same shuttle components were also shown to function as Mal/2-OG translocators in vitro [296]. This represents another variation to shuttle organellar Mal in exchange for cytosolic 2-OG. Finally, via low-carbon-inducible 20 (LCI20) [295], *Chlamydomonas reinhardtii* may also directly reimport Mal into the chloroplast in exchange for Glu. LCI20 in conjunction with Mal/2-OG translocators could partake in a zero-sum Mal exchange with the cytosol. Accordingly, 2-OG is imported into the chloroplast as a carbon skeleton for the plastidial FDX-dependent glutamine 2-oxoglutarate aminotransferase. The activity of the latter sustains photosynthetic electron transfer by oxidizing the PSI electron acceptor pool, and producing Glu which can then be exported into the cytosol. Interestingly, Mal trafficking is crossing photorespiration pathways on multiple occasions, thereby helping to convert glyoxylate into Gly, but it might be nuanced in terms of organellar routes depending on the organism. In conclusion, the Mal shuttle helps to keep up photosynthesis by adjusting the ATP/NADPH ratio in chloroplasts. Although our detailed understanding of the process is built on a multitude of land plant studies, recent works from diatoms [229] and green algae [297] show that the Mal shuttle is actively contributing to photosynthetic fitness even in organisms that possess CCMs to keep photorespiration rates low. CCMs and photorespiratory bypass strategies are only two good examples of how microalgae inspire current research approaches to improve photosynthesis in land plants [298].

## 6. Ion Conductivity Regulation to Optimize ATP Yields

### 6.1. ATP Synthase Regulation

As outlined above, the pigment-containing complexes participating in the capture and conversion of light energy into chemical energy display various fine-tuning features to match electron transfer rates with the metabolic capacity of the cell. This fine-tuning determines the competence to generate the light-driven *pmf*. The latter is an electrochemical gradient across the photosynthetic membrane and here, we will focus on mechanisms that regulate ion conductivity to optimize ATP yields. The F_O_F_1_-ATP synthase (F_O_F_1_) is an ancient enzyme that predates photosynthesis (and possibly electron transfer chains), since it took over a fundamental role during early evolution of cellular bioenergetics [299]. F_O_F_1_ in the photosynthetic membrane of the green lineage [300] and diatoms [301] follows a simple architecture that resembles its eubacterial counterpart; it does not fulfill an ultrastructural role as the homolog in eukaryotic oxidative respiration [302]. However, the primary bioenergetic function of F_O_F_1_ is conserved: It matches the energies stored as phosphorylation potential with the one stored as *pmf*. Accordingly, reversible ADP phosphorylation is carried out by the soluble F_1_ part in the photosynthetic cell compartment in a reaction governed by the concentration ratio of nucleotides and inorganic phosphate, i.e., ([ATP])/([ADP][P_i_]). A high *pmf* will drive H^+^ passage from the lumen into the stroma to yield ATP; H^+^ will be pumped into the lumen when ATP levels are high and/or the *pmf* is low. F_1_ is a chemical motor composed of stochiometric subunits α_3_β_3_γ_1_δ_1_ε_1_ being mechanically coupled to the electrochemical motor F_O_ (composed of a_1_b_1_b’_1_c*_n_* subunits, also called I_1_II_1_III_n_IV_1_) that translocates *n* H^+^ ions during a full rotation to form/hydrolyze 3 ATP molecules. In photosynthetic membranes, a certain flexibility is seen when it comes to the H^+^/ATP ratio imposed by the oligomerization state of the F_O_ subunit c, leaning towards larger ratios when compared to respiratory membranes. Hence, ATP synthesis can be catalyzed at relatively low *pmf* levels and moderate lumen acidification [303]. Compared to the c_14_ oligomer in vascular plants [304], there is preliminary evidence that c_13_ exists in *Chlamydomonas reinhardtii* [305] and the requirement to elicit the pH response of Cyt*b_6_f* and NPQ under more acidic lumen conditions [173,174] might point to even smaller c rings in diatoms. Evidently, F_O_F_1_ is the major H^+^ gate in photosynthesis and various environmental stimuli shift the enzyme’s activation energy. This in turn influences the intricate relationship F_O_F_1_ shares with the *pmf*, the master regulator of photosynthesis. NPQ and photosynthetic control play a major protective role in response to ΔpH and under high light conditions, these processes may be facilitated by a slowdown of H^+^ translocation activity via F_O_F_1_. Such a slowdown of H^+^ translocation upon high light has been shown in vascular plants [306] and green algae [307] but insights from other aquatic species are currently missing. Other environmental stimuli associated with land plant F_O_F_1_ downregulation are cold temperatures [308] or low CO_2_ levels [309] but, again, studies of F_O_F_1_ from aquatic phototrophs under those conditions are scarce. In either case, F_O_F_1_ activity-tuning results from the fact that the carbon metabolism is influenced by the environmental condition. Since the light intensity can easily exceed the energy conversion capacity under those restricted metabolic conditions, the need to regulate light-harvesting efficiencies and electron transfer rates is obvious (see Section 2.3 and Section 3.1).

### 6.2. Ion Channels for pmf Parsing

Over the last decades, more insights and concepts on *pmf* parsing, the fine-tuning of ΔΨ and ΔpH, were postulated [310]. Here, we will briefly cover a selection of channels and antiporters in the thylakoid membrane of microalgae (extensively reviewed in [311]) with immediate impact on the *pmf*. This excludes certain antiporters, such as triose phosphate/phosphate translocators that ensure optimal photo-assimilate exchange [312] and Mal valve-related processes that have been mentioned in Section 5.2. Moreover, ion-conducting proteins in the inner chloroplast envelope will not be covered here as the stromal space is substantially larger than the lumen volume. One of the major proteins in the context of *pmf* parsing is KEA3, the luminal H^+^/stromal K^+^ antiporter that converts ΔpH for ΔΨ in land plants [313,314] and diatoms [174]. KEA3 is also encoded in other microalgae, except for Glaucophytes [315], but functional studies are missing. By consuming ΔpH, KEA3 was shown to be important for NPQ relaxation during light intensity transitions and *kea3* mutants usually show excessive NPQ. The H^+^/K^+^ antiporter from plants is supposedly tweaked by stromal nucleotides and NAD(P)H via its C-terminal domain [316,317]. This domain takes over a similar role in diatoms, but there it contains an EF-hand motif to bind Ca^2+^ [174]. This intriguing fine-tuning connects H^+^/K^+^ antiport activity with the physiological state of the photosynthetic cell as levels of Ca^2+^, NAD(P)H, and ATP are variable throughout the day. Another example of fine-tuning *pmf* parsing occurs via voltage-dependent anion channels. Here, photosynthesis-driven ΔΨ, which varies throughout the day, would trigger channel activation. Two types of Cl^-^ channels (CLC) are known to participate: CLCe members of the CLC family [318,319] and VCCN members of a new family type [320,321]. The latter have been investigated in *Arabidopsis thaliana* to influence the *pmf* by dissipating ΔΨ in favor of ΔpH to induce NPQ. Although homologs exist in microalgae [295,322] functional characterization data on CLCe [323,324] and VCCN are limited in the literature. On top of that, CCM-related ion transporters have also shown to impact *pmf* formation per se via the reversible protonation of CO_2_ upon its passage into the lumen [325].

## 7. Concluding Remarks

The photosynthetic apparatus is a sophisticated and intertwined machinery that maintains efficient energy conversion rates under varying environmental conditions. In this review, we covered the basic blueprint of how photosynthetic electron transfer generates the *pmf* and how fine-tuning the latter is pivotal for survival in an everchanging surrounding. We highlighted several special adaptations of oxygenic photosynthesis in microalgal systems. Besides already exploited feats such as sourcing lipid-rich biomass, the microalgal group in its yet to be fully explored diversity holds promising photoprotective traits that may be beneficial for photosynthesis in the field. Assembled data from different niches should therefore hold a key constituent for future studies, which could pave the road for bioengineering a more resistant, adaptable, and efficient system.

## Data Availability

No new data were created or analyzed in this study.
